# C-reactive protein-to-lymphocyte ratio and trabecular bone score: A mediation analysis of BMI in the NHANES 2005 to 2008 cohort

**DOI:** 10.1097/MD.0000000000043847

**Published:** 2025-08-08

**Authors:** Zhizhuang Wang, Bo Xu, Guoliang Ma, Xin Chen, Bowen Yang, Xiaokuan Qin, Weili Duan, Minshan Feng, Kai Sun, Liguo Zhu, He Yin

**Affiliations:** a Department of Spine, Wangjing Hospital, China Academy of Chinese Medical Sciences, Beijing, China; b Nanyang Hospital, Wangjing Hospital, Chinese Academy of Traditional Chinese Medicine (Dushan Hospital District), Henan, China; c Nanyang Key Laboratory of Orthopedic Biomechanics of Traditional Chinese Medicine, Henan, China; d Beijing Key Laboratory of Bone Setting Technology of Traditional Chinese Medicine, Beijing, China.

**Keywords:** body mass index, C-reactive protein, C-reactive protein-to-lymphocyte ratio, osteoporosis, Trabecular Bone Score

## Abstract

Previous studies have focused on the relationship between C-reactive protein and Trabecular Bone Score (TBS), but the correlation between C-reactive protein-to-lymphocyte ratio (CLR) and TBS remains unclear. Some studies have shown that of body mass index (BMI) is associated with CLR and TBS, respectively. This study focused on the correlation between CLR and TBS and the mediating effect of BMI based on data from the National Health and Nutrition Examination Survey (NHANES) from 2005 to 2008. This study used data from the NHANES database (1789 males and 1699 females) to perform multivariate logistic regression, population characterization, and subgroup and interaction analyses to estimate the relationship between CLR and TBS, and explored the mediating role of BMI on the correlation between CLR and TBS through mediation analysis. A total of 3488 participants aged 20 years and older were recruited for this study. Multivariate regression analysis showed that those with a high CLR had a low TBS. Mediation analyses showed that CLR had a large direct effect on TBS (*P* < .0001) and that BMI partially mediated this relationship (55.2148%, *P* < .0001). This study found that increased levels of CLR were associated with decreased TBS and were influenced by BMI. CLR may be a valuable tool for assessing bone metabolism and deserves further exploration. These findings contribute to a deeper understanding of the impact of obesity on the lineage of the relationship between inflammation and bone health and provide new perspectives on the treatment and prevention of osteoporosis and osteoporotic fractures.

## 1. Introduction

Osteoporosis is a long-term, progressive systemic bone disease characterized by diminished bone mineral density (BMD) and degradation of skeletal microarchitecture, which significantly expands the risk associated with fracture and exerts a severe effect on the physical and mental well-being of patients.^[1,2]^ Osteoporotic fractures represent a major complication of osteoporosis, often associated with increased mortality and disability rates. These fractures not only significantly impair patients’ health and the level of well-being and standard of living but also impose a significant financial encumbrance upon society.^[3–5]^ According to U.S. statistics from the population count, in 2010, approximately 10.2 million older adults were estimated to have osteoporosis, with another 43.4 million people presenting with low BMD, placing them at an elevated risk of osteoporotic bone fractures.^[6]^ BMD is frequently utilized for the diagnosis of osteoporosis. While BMD can pinpoint numerous people with a high risk, relying solely on BMD for bone break risk assessment might be inadequate, particularly in individuals having BMD that is either normal or elevated.^[7]^

Trabecular bone score represents an innovative approach that assesses bone quality by analyzing gray-scale characteristics of lumbar vertebrae spine dual-energy X-ray absorptiometry images.^[8]^ In contrast to BMD, TBS can identify individuals with deteriorated bone microarchitecture despite having normal BMD levels and provides a more accurate prediction of osteoporotic fracture risk.^[9,10]^ TBS provides a more effective assessment of fracture risk in individuals with normal or slightly diminished BMD who might be susceptible to risk for fractures caused by minimal force, as well as in high-risk individuals with already compromised bone microarchitecture.^[11]^ Consequently, TBS has accumulated considerable attention as a valuable indicator of bone microarchitecture and overall bone quality.

C-reactive protein-to-lymphocyte ratio (CLR) has emerged as a new type of inflammation indicator reflecting the balance between systemic inflammation and immune response.^[12]^ The CLR, derived by dividing C-reactive protein (CRP) by lymphocyte count, combines these 2 parameters to provide a more comprehensive measure of inflammatory status, with higher values indicating a pro-inflammatory state.^[13]^ Recent studies have demonstrated the clinical relevance of CLR across various conditions, such as cardiovascular disease,^[14]^ cancer prognosis,^[15]^ and obesity,^[16]^ suggesting that it may serve as a practical marker for evaluating systemic inflammation and immune balance in clinical and research settings. While several investigations have explored the correlation between CRP and TBS,^[17]^ few have probed the linkage between CLR and TBS. Meanwhile, BMI is a core indicator of nutritional status and metabolic health, and has a complex relationship with bone health.^[18]^ On the one hand, high BMI may have a protective effect on bone by increasing body weight load^[19]^; on the other hand, excessive adiposity may lead to chronic low-grade inflammation, which may negatively affect bone health.^[20]^ Whether BMI plays a mediating role in the relationship between CLR and TBS, as well as the specific mechanism of this mediating effect, has not yet been clarified. To address this knowledge gap, we analyzed statistics from the 2005 to 2008 National Health and Nutrition Examination Survey (NHANES). Using rigorous inclusion criteria and adjustments for relevant covariates, we set out to elucidate the relationship between CLR levels and TBS as well as the mediating role of BMI in the relationship between CLR and TBS, revealing potential mechanisms underlying the relationship between inflammation, nutritional status, and bone trabecular microarchitecture.

## 2. Methods

### 2.1. Study design and population

**Figure 1.**
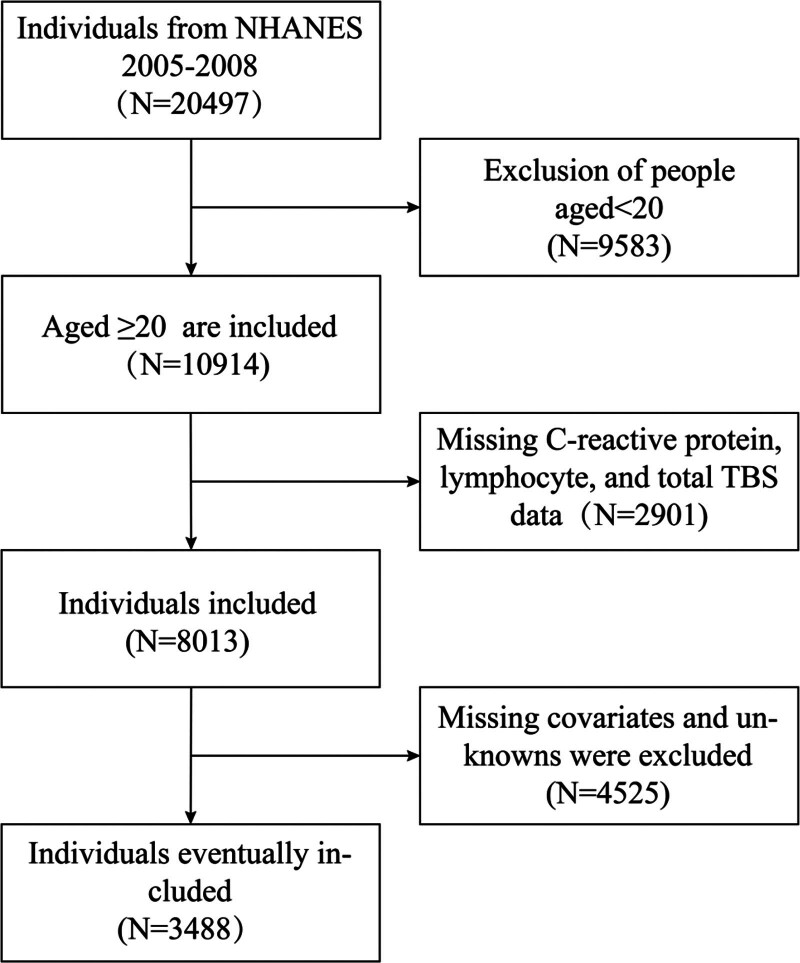
Flow chart of participants selection from the NHANES 2005 to 2008. N = number, NHANES = National Health and Nutrition Examination Survey, TBS = trabecular bone score.

NHANES is a cross-sectional analysis with national representativeness, purposefully structured to comprehensively evaluate the health and nutritional profile of the United States population.^[21]^ NHANES database encompasses a wide range of data, including demographic information, bodily measurements, survey replies, and lab analysis outcomes, collected through sophisticated sampling methodologies.^[22]^ Comprehensive NHANES data are publicly available online and have been approved by the Ethics Review Board of the National Center for Health Statistics.^[23]^ Written informed consent was secured from all participants prior to their involvement in the study. Data analysis was undertaken from April 1 to April 30, 2024. Details on the National Center for Health Statistics IRB/ERB Protocol Number are available in the Supplementary Material, Supplemental Digital Content, https://links.lww.com/MD/P643. In this cross-sectional investigation, we initially identified 20,497 individuals from the 2005 to 2008 NHANES dataset, and after applying stringent inclusion and exclusion criteria, we finalized a cohort of 3488 participants. The detailed selection process is illustrated in Figure 1. Exclusion criteria included: (1) individuals under 20 years of age, (2) participants with missing data on TBS, CRP, and lymphocytes, and (3) individuals with incomplete or ambiguous data on other variables. The screening process can be defined as follows: it involves the steps of the assessment routine (Fig. 1).

### 2.2. Predictor variable

In this study, the independent variable, CLR, was calculated by dividing the serum concentration of CRP by the absolute lymphocyte count. CRP levels were quantified using latex-enhanced turbidimetry, with signal data reduction performed via a storable logit-log function applied to the calibration curve. Quantitative CRP was quantified utilizing a Behring nephelometer for turbidimetric analysis. Lymphocyte counts, rather than percentages, were used to derive CLR. Also, for statistical analysis, CLR levels within the context of this investigation were systematically classified into 4 clusters according to quartiles: first quartile (Q1): 0.00 to 0.04, second quartile (Q2): 0.04 to 0.10, third quartile (Q3): 0.10 to 0.23, and fourth quartile (Q4): 0.23 to 11.76.

### 2.3. Outcome variable

TBS was the outcome variable in this study, serving as a surrogate index of bone texture. TBS was assessed by analyzing gray-scale variations in the pixel distribution of lumbar spine dual-energy X-ray absorptiometry scans. These scans were acquired using a Hologic QDR-4500A fan-beam densitometer (Hologic, Inc., Bedford, MA). TBS values were subsequently computed using TBS software (Med-Imap SA TBS Calculator, version 2.1.0.2) for participants aged 20 years and older.

### 2.4. Assessment of mediator

In this study, BMI was considered as the mediating factor. BMI is a simple calculation based on a person’s weight in kilograms divided by the square of their height in meters.

### 2.5. Assessment of covariates

In view of antecedent scholarly inquiries and clinical insights,^[24–26]^ we included key covariates in our analysis to account for potential confounders in the interplay between CLR and TBS. Continuous covariates included age, household poverty-to-income ratio (PIR), serum albumin (g/dL), alanine aminotransferase (U/L), aspartate aminotransferase (U/L), blood urea nitrogen (mg/dL), total calcium (mg/dL), cholesterol (mg/dL), total protein (g/dL), high-density lipoprotein cholesterol (HDL-C) (mg/dL), low-density lipoprotein cholesterol (mg/dL), uric acid (mg/dL), and hemoglobin (g/dL). Categorical covariates were sex, age group, race/ethnicity, educational attainment, PIR, and smoking status (characterized by a history of consuming a minimum of 100 cigarettes over the course of an individual’s lifetime).

### 2.6. Statistical analysis

Prior to commencing the statistical examination, the combined dataset was assessed for normality to comply with the requirements of this study. For continuous variables, this was done by applying a weighted logistic regression model, while for categorical variables, the analysis used a weighted chi-square test to assess correlation. Continuous variables were expressed as means with standard deviation as a measure of variability, and categorical variables were described by proportional frequencies. To investigate the relationship between CLR and TBS, weighted multivariate logistic regression analyses were conducted for the following 3 models: model 1, unadjusted model; model 2, adjusted for sex, age, and race/ethnicity; and model 3, adjusted for full covariates. Meanwhile, causal mediating effects analysis was used to identify possible mediating effects of BMI on the relationship between CLR and TBS. To further investigate potential interactions and to account for confounders in categorical variables, subgroup analyses were performed and the results visualized.

All computations were executed with the utilization of R (http://www.R-project.org) and EmpowerStats software (http://www.empowerstats.com). Upon achieving a *P*-value of <.05 for a two-tailed test, the result is deemed statistically significant.

## 3. Results

### 3.1. Characteristics of the participants

There were 3488 subjects aged 20 years or above in this study, with a mean age of 46.54 ± 16.11 years. In the demographic and clinical profiling of participants, data were analyzed with stratification by CLR quartiles. We observed significant differences between CLR quartiles for several variables. First, age, PIR, albumin, total calcium, total cholesterol, uric acid, hemoglobin, HDL-C, low-density lipoprotein cholesterol, and TBS showed statistically significant differences (*P* < .0001). As CLR increased, age and uric acid levels increased, while albumin, total calcium, hemoglobin, HDL-C, and TBS gradually decreased, suggesting that these metrics may be associated with inflammation levels. In addition, sex, race/ethnicity, and education level differed significantly across CLR quartiles, with females, African Americans, and those with lower levels of education being more prevalent in the higher CLR quartiles. In contrast, alanine aminotransferase, aspartate aminotransferase, blood urea nitrogen, and total protein did not show significant differences between CLR quartiles (*P* > .05), indicating that these metrics may not be significantly associated with CLR. In addition, no significant differences in smoking status were observed among the participants stratified by CLR quartiles (*P* = .2979) (Table 1).

**Table 1:** Characteristics of the study population based on CLR quartiles.

Characteristic	C-reactive protein-to-lymphocyte ratio (CLR)					*P*-value
	Total	Q1 (0.00–0.04)	Q2 (0.04–0.10)	Q3 (0.10–0.23)	Q4 (0.23–11.76)	
N	3488	869	872	875	872	
Age (yr)	46.54 ± 16.11	41.72 ± 15.49	46.74 ± 15.78	48.73 ± 15.99	49.83 ± 15.97	<.0001
PIR (%)	3.17 ± 1.59	3.34 ± 1.56	3.23 ± 1.59	3.10 ± 1.62	2.96 ± 1.58	<.0001
Albumin (g/dL)	4.24 ± 0.31	4.37 ± 0.28	4.29 ± 0.30	4.21 ± 0.28	4.07 ± 0.31	<.0001
ALT (U/L)	25.66 ± 19.26	24.20 ± 13.50	26.30 ± 18.06	26.09 ± 15.47	26.24 ± 28.01	.0538
AST (U/L)	25.33 ± 16.34	24.69 ± 9.55	25.43 ± 11.78	25.41 ± 10.09	25.91 ± 28.22	.4727
Blood urea nitrogen (mg/dL)	12.69 ± 5.01	12.38 ± 4.17	12.98 ± 4.92	12.62 ± 4.91	12.83 ± 6.02	.0574
Total calcium (mg/dL)	9.40 ± 0.34	9.45 ± 0.33	9.42 ± 0.34	9.40 ± 0.35	9.33 ± 0.34	<.0001
Cholesterol (mg/dL)	198.13 ± 39.99	193.32 ± 37.45	196.86 ± 36.56	202.87 ± 43.34	200.37 ± 42.17	<.0001
Total protein (g/dL)	7.13 ± 0.44	7.13 ± 0.45	7.11 ± 0.45	7.13 ± 0.44	7.14 ± 0.44	.6479
Uric acid (mg/dL)	5.50 ± 1.34	5.21 ± 1.29	5.46 ± 1.28	5.58 ± 1.34	5.79 ± 1.39	<.0001
Hemoglobin (g/dL)	14.56 ± 1.49	14.70 ± 1.45	14.74 ± 1.44	14.56 ± 1.53	14.18 ± 1.50	<.0001
HDL-C (mg/dL)	54.72 ± 15.29	57.97 ± 16.38	55.00 ± 14.96	53.19 ± 14.23	52.08 ± 14.65	<.0001
LDL-C (mg/dL)	116.42 ± 35.17	111.48 ± 32.30	115.73 ± 32.54	120.88 ± 38.58	118.43 ± 36.74	<.0001
TBS	1.39 ± 0.14	1.45 ± 0.12	1.40 ± 0.13	1.37 ± 0.14	1.32 ± 0.16	<.0001
Sex						<.0001
Male	49.65	56.81	53.35	47.57	39.08	
Female	50.35	43.19	46.65	52.43	60.92	
Race/ethnicity						<.0001
Mexican American	7.76	7.20	7.96	8.09	7.85	
Other Hispanic	3.82	2.85	4.88	4.32	3.30	
Non-Hispanic White	72.46	71.03	73.57	72.54	72.85	
Non-Hispanic Black	10.37	9.61	8.46	10.51	13.25	
Other race	5.59	9.31	5.13	4.53	2.75	
Education						<.0001
<9th grade	5.91	4.87	6.02	7.03	5.86	
9–11th grade (includes 12th grade with no diploma)	11.66	9.77	12.30	10.53	14.42	
High school grade/GED or equivalent	24.61	21.10	25.08	26.04	26.80	
Some college or AA degree	30.05	30.97	24.86	30.57	34.18	
College graduate or above	27.77	33.29	31.73	25.83	18.74	
Smoke (%)						.2979
Yes	49.49	46.94	51.11	50.23	49.95	
No	50.51	53.06	48.89	49.77	50.05	

Mean ± SD for continuous variables: the *P*-value was calculated by the weighted linear regression model. (percentage) For categorical variables, the *P*-value was calculated by the weighted chi-square test.

ALT = alanine aminotransferase, AST = aspartate aminotransferase, CLR = C-reactive protein-to-lymphocyte ratio, HDL-C = high-density lipoprotein cholesterol, LDL-C = low-density lipoprotein cholesterol, N = number, PIR = household poverty-to-income ratio, TBS = trabecular bone score.

### 3.2. Association between CLR and TBS

We assessed the relationship between CLR and TBS by analyzing CLR as a continuous variable and quartiles using a multivariate logistic regression model (Table 2). The analysis revealed that CLR exhibited a robust negative association with TBS and that this negative correlation persisted after adjusting for different covariates. In Model 1, a statistically significant decline was noted in TBS for each 1-unit increase in CLR (β = −0.27, 95% CI: −0.30, −0.24, *P* < .0001). Upon accounting for sex, age, and race in Model 2, the detected correlation was slightly attenuated (β = −0.22, 95% CI: −0.24, −0.19, *P* < .0001). In Model 3, with all covariates adjusted for, the association weakened to β = −0.13 (95% CI: −0.16, −0.10, *P* < .0001), but continued to exhibit statistical significance. A similar trend was observed when we analyzed CLR as a categorical variable in quartiles. Contrasted against the reference group (Q1), the β-value for TBS was progressively lower from Q2 to Q4 in all models, suggesting that higher CLR levels were inversely associated with TBS. Specifically, in Model 1, the β values for the Q2, Q3, and Q4 quartiles were −0.05, −0.08, and −0.12, respectively (all *P* < .0001). These values decreased to −0.03, −0.05, and −0.09 in Model 2 and further decreased to −0.02, −0.03, and −0.06 in Model 3, but remained significant. The *P*-value for the trend was consistently <.0001 in all models, which reinforces the negative trend between CLR and TBS. After converting CLR to categorical variables (quartiles), the CLR for the top quartile was 0.06 lower than that for the lowest quartile. Also, the trend tests all had *P* < .001, demonstrating a significant declining direction in TBS with increasing levels of CLR. This pattern suggests that greater tiers of CLR are connected with decreased TBS, and this association remains even after multivariate adjustment.

**Table 2:** Association of TBS with CLR in different models among all participants.

	Model 1 β (95% CI)	*P*-value	Model 2 β (95% CI)	*P*-value	Model 3 β (95% CI)	*P*-value
CLR per 1 unit increase	−0.27 (−0.30, −0.24)	<.0001	−0.22 (−0.24, −0.19)	<.0001	−0.13 (−0.16, −0.10)	<.0001
CLR (quartile)						
Q1 (0.00–0.04)	Ref.		Ref.		Ref.	
Q2 (0.04–0.10)	−0.05 (−0.06, −0.04)	<.0001	−0.03 (−0.04, −0.02)	<.0001	-0.02 (-0.03, −0.00)	.0047
Q3 (0.10–0.23)	−0.08 (−0.09, −0.07)	<.0001	−0.05 (−0.07, −0.04)	<.0001	-0.03 (-0.04, −0.02)	<.0001
Q4 (0.23–11.76)	−0.12 (−0.14, −0.11)	<.0001	−0.09 (−0.11, −0.08)	<.0001	-0.06 (-0.07, −0.04)	<.0001
*P* for trend	<.0001		<.0001		<.0001	

Model 1: no covariates were adjusted. Model 2: age, sex, and race/ethnicity were adjusted. Model 3: sex, age, race/ethnicity, education level, PIR, serum albumin, ALT, AST, blood urea nitrogen, total calcium, cholesterol, total protein, HDL-C, LDL-C, uric acid, hemoglobin, and smoking status were adjusted.

CLR = C-reactive protein-to-lymphocyte ratio, TBS = trabecular bone score.

### 3.3. Subgroup analysis

**Figure 2.**
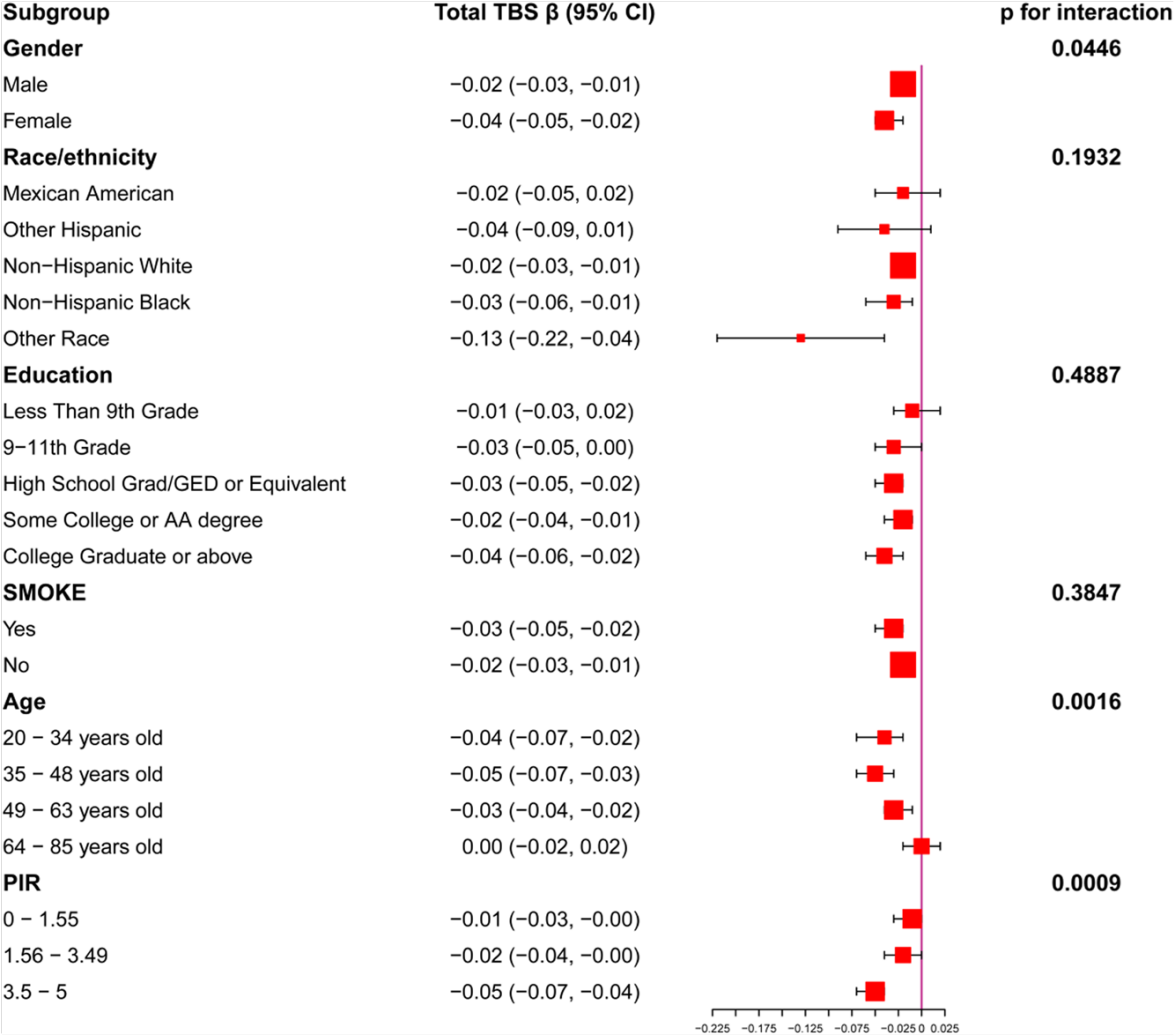
Association between CLR and TBS according to subgroup. The model adjusts all variables except CLR, TBS, and stratification variables. PIR = household poverty-to-income ratio, TBS = trabecular bone score.

In this study, we performed subgroup-specific analyses to explore the robustness of the correlation between CLR and TBS in different demographic contexts. The results of subgroup analysis were also visualized through pictures (Fig. 2). In the sex subgroup, the negative correlation of CLR on TBS was more significant in women compared to men (men: β = −0.02, 95% CI: −0.03, −0.01; women: β = −0.04, 95% CI: −0.05, −0.02; *p* for interaction = 0.0446). Analyses of race/ethnicity subgroups failed to display a substantial interaction (*P* for interaction = .1932), but the strongest negative association between CLR and TBS was found in the “other ethnicity” category (β = −0.13, 95% CI: −0.22, −0.04). The education subgroup demonstrated that the relationship between CLR and TBS stood out more among those with higher levels of education (*P* for interaction = .4887), especially those who had graduated from university and above (β = −0.04, 95% CI: −0.06, −0.02). Within the smoking status subset, no discernible change was seen between smokers and nonsmokers (*P* for interaction = .3847). Of note, there was a noteworthy interaction in the age (*P* for interaction = .0016) and PIR (*P* for interaction = .0009) subgroups. Age analysis demonstrated that the adverse correlation between CLR and TBS was strongest in the 20 to 48-year-old group, and this association gradually weakened with age, whereas no significant association was shown in those aged 64 years or older. PIR analysis demonstrated that the adverse link between CLR and TBS was strongest in the high-income group (PIR 3.5–5) (β = −0.05, 95% CI: −0.07, −0.04). Sex, age, and PIR significantly influenced the affiliation between CLR and TBS, whereas the other subgroups revealed no meaningful interaction (Table 3).

**Table 3:** Relationship between CLR and TBS analyzed according to subgroups.

Subgroup	TBS β (95% CI)	*P* for interaction
Gender		.0446
Male	−0.02 (−0.03, −0.01)	
Female	−0.04 (−0.05, −0.02)	
Race/ethnicity		.1932
Mexican American	−0.02 (−0.05, 0.02)	
Other Hispanic	−0.04 (−0.09, 0.01)	
Non-Hispanic White	−0.02 (−0.03, −0.01)	
Non-Hispanic Black	−0.03 (−0.06, −0.01)	
Other Race	−0.13 (-0.22, −0.04)	
Education		.4887
<9th Grade	−0.01 (-0.03, 0.02)	
9–11th Grade (includes 12th grade with no diploma)	−0.03 (−0.05, 0.00)	
High school grade/GED or equivalent	−0.03 (−0.05, −0.02)	
Some college or AA degree	−0.02 (−0.04, −0.01)	
College graduate or above	−0.04 (−0.06, −0.02)	
Smoke		.3847
Yes	−0.03 (−0.05, −0.02)	
No	−0.02 (−0.03, −0.01)	
Age		.0016
20–34 years old	−0.04 (−0.07, −0.02)	
35–48 years old	−0.05 (−0.07, −0.03)	
49–63 years old	−0.03 (−0.04, −0.01)	
64–85 years old	0.00 (−0.02, 0.02)	
PIR		.0009
0–1.55	−0.01 (−0.03, −0.00)	
1.56–3.49	−0.02 (−0.04, −0.00)	
3.5–5	−0.05 (−0.07, −0.04)	

This model adjusted for all variables except CLR, total TBS, and corresponding stratified variables.

CLR = C-reactive protein-to-lymphocyte ratio, PIR = household poverty-to-income ratio, TBS = trabecular bone score.

### 3.4. Causal mediation analysis

**Figure 3.**
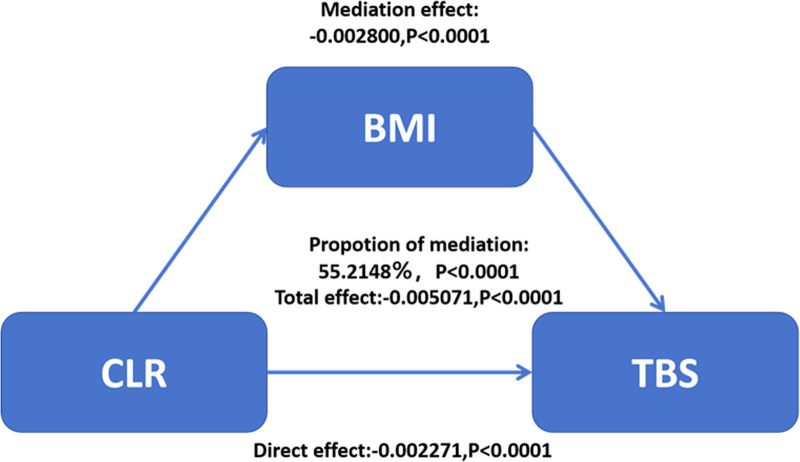
Causal mediation analysis of BMI in the relationship between CLR and TBS. BMI = body mass index, CLR = C-reactive protein-to-lymphocyte ratio, TBS = trabecular bone score.

This mediation analysis revealed a significant negative association between CLR and TBS, with BMI playing a substantial mediating role. The total effect of CLR on TBS was −0.005071 (95% CI: −0.010056 to −0.004085, *P* < .0001), of which 55.2% was mediated through BMI (−0.002800, 95% CI: −0.006752 to −0.001702, *P* < .0001). The direct effect of CLR on TBS, independent of BMI, remained significant (−0.002271, 95% CI: −0.004389 to −0.001340, *P* < .0001) (Fig. 3, Table 4).

**Table 4:** BMI as a mediator of the relationship between CLR and TBS.

Mediation effect (CLR–BMI–TBS)	Estimate	95% CI lower	95% CI upper	*P*-value
Total effect	-0.005071	-0.010056	-0.004085	<.0001
Mediation effect	-0.002800	-0.006752	-0.001702	<.0001
Direct effect	-0.002271	-0.004389	-0.001340	<.0001
Proportion mediated	0.552148	0.370233	0.776379	<.0001

Sex, age, race/ethnicity, education level, PIR, serum albumin, ALT, AST, blood urea nitrogen, total calcium, cholesterol, total protein, HDL-C, LDL-C, uric acid, hemoglobin, and smoking status were adjusted. Proportion of mediation = Mediation effect/Direct effect + Mediation effect; Total effect = Direct effect + Mediation effect.

BMI = body mass index, CLR = C-reactive protein-to-lymphocyte ratio, TBS = trabecular bone score.

## 4. Discussion

This investigation explored the BMI-mediated relationship between CLR and TBS by examining health information from the NHANES database for people aged 20 years and older from 2005 through 2008. Overall, there was a significant negative correlation between CLR and TBS. Notably, in the sex subgroup, females showed a stronger negative correlation between CLR and TBS, while in the age subgroup, the negative correlation was more significant between 35 and 48 years. Analyzing the possible reasons for the above, women may be more sensitive to chronic low-grade inflammation (e.g., inflammatory state expressed through high CLR) due to the complexity of the endocrine system. Inflammatory factors (e.g., IL-6 and TNF-α) promote osteoclast activity and inhibit osteoblast function, leading to a decrease in bone mass and a lowering of TBS.^[27]^ At the same time, menopausal women face a period of rapid bone loss, and TBS in particular is more susceptible to inflammation-driven bone resorption.^[28–30]^ This may make the negative correlation between CLR and TBS more pronounced in women. In the PIR subgroup, we still observed significant negative correlations, a situation that could be explained by several mechanisms. For example, populations with lower PIR usually face nutritional deficiencies, such as insufficient intake of key nutrients such as calcium, vitamin D, and protein, which are essential for maintaining bone health.^[31]^ Poor nutrition affects the process of bone formation and accelerates bone resorption, thereby decreasing TBS.^[32]^ In addition, unhealthy dietary habits (e.g., high-fat, high-sugar diets) may exacerbate the chronic inflammatory state, further amplifying the negative effects of CLR on TBS. Individuals with a lower PIR are more likely to live in food swamps, and deficiencies in antioxidant-acting micronutrients (e.g., zinc, selenium, etc) may lead to elevated levels of oxidative stress, which in turn promotes increased CRP levels and amplifies the negative correlation between CLR and TBS.^[33,34]^ Race/ethnicity, education level, and smoking status did not reach significant levels of interaction, although they showed negative associations. Based on intermediation analysis, our study suggests that individuals with a higher BMI may exhibit lower TBS. Based on these findings, we went on to conduct a mediation analysis. We found that BMI had a significant impact on the relationship between CLR and TBS.BMI partially mediated the relationship between high CLR and low TBS. The mediation proportion of the variable was 55.2148%. In recent years, researchers have conducted several studies on the relationship between BMI and TBS. Some studies have shown that there is a significant negative correlation between BMI and TBS.^[35,36]^ The results of the mediation analysis in this study support this view. This mediating effect can be explained by several mechanisms. For example, BMI is widely used as a standardized index to determine the degree of obesity in an individual, which is strongly associated with chronic systemic inflammation.^[37]^ Elevated BMI is often accompanied by an expansion of adipose tissue, especially the accumulation of visceral fat, which leads to an excessive release of pro-inflammatory cytokines (e.g., TNF-α, IL-6).^[38]^ These cytokines activate the liver to synthesize more CRP, which leads to elevated CLR levels.^[39]^ In contrast, a chronic inflammatory state accelerates the process of bone resorption, inhibits bone formation, and directly affects the quality and quantity of trabecular bone structures, thereby reducing TBS.^[26]^ Obesity-associated adipose tissue not only releases pro-inflammatory factors, but also leptin and adiponectin via paracrine pathways.^[40]^ Leptin promotes bone resorption through the central nervous system, whereas adiponectin may exert some protective effects on bone metabolism through its anti-inflammatory properties.^[41]^ Elevated BMI may lead to excessive increases in leptin levels and decreases in adiponectin levels, which may adversely affect TBS.^[42]^

CRP is a classical indicator of inflammation commonly utilized to keep an eye on infection and circumstances that cause inflammation.^[43]^ Lymphocytes, an important component of the immune response, are usually reduced in number during excessive immune activation.^[44]^ In clinical practice, leukocytes, neutrophils, lymphocytes, platelets, and CRP are often used as markers of inflammation. Using a mix of these indicators for inflammation is often more reproducible and accurate than a single indicator. In the last several years, a rising number of studies have shown that CLR, an emerging inflammatory marker, can be better used to predict disease prognosis and for diagnostic evaluation.^[13,45]^ Our study showed a significant negative correlation between CLR and TBS unadjusted and fully adjusted for covariates, which persisted when mediated by BMI. This suggests that chronic inflammation may contribute to the deterioration of skeletal microstructure. First, heightened CLR reflects chronic low-grade inflammation, which promotes osteoclast activity and inhibits osteoblast function, leading to an imbalance in bone metabolism.^[46]^ Second, lymphopenia may indicate impaired immune function, and immune disorders are closely related to the bone remodeling process.^[47]^ In addition, oxidative stress and altered skeletal microenvironment accompanying chronic inflammation and immune abnormalities may further accelerate bone loss.^[48]^ Ultimately, inflammation and metabolic abnormalities may affect the metabolism of key nutrients for the health of bones, such as calcium and vitamin D, thereby increasing the risk of osteoporosis.^[49]^

In recent years, the correlation between bone health and inflammation has attracted much scholarly scrutiny.^[50,51]^ Our findings suggest a strong inverse relationship between CLR and TBS and are influenced by BMI. The study suggests that excessive obesity may lead to the development of chronic inflammation, which can lead to the risk of elevated CLR, which in turn decreases TBS values and increases the risk of osteoporosis. Keeping BMI within a moderate range, reducing inflammation production, lowering CLR values and increasing the level of TBS values can help to further deter osteoporotic fractures and provide recommendations for clinical application.

This study has a number of strengths. First, this is the first study to mediate the relationship between CLR levels and TBS via BMI. Second, we employed a large sample of data and used multivariate adjustment models to adjust for potential confounders. In addition, we comprehensively assessed the effect of BMI-mediated CLR levels on TBS through multivariate logistic regression, subgroup analyses, and causal mediated effects analysis. However, it is important to recognize certain limitations of this study. First, as a cross-sectional investigation, we were unable to determine a causal relationship between CLR level and TBS. Second, most of the study participants were from the United States, and it is unclear whether the findings apply to other countries or regions. In addition, lifestyle and nutritional differences may also have an impact on CLR levels. Finally, we acknowledge BMI as a standardized indicator of an individual’s obesity level, but BMI has limitations in accurately reflecting fat distribution. More comprehensive metrics are needed to better assess an individual’s bone health.

## 5. Conclusion

In summary, this study confirmed that there was a significant negative correlation between CLR levels and TBS, and that increased CLR was associated with decreased TBS. Furthermore, it suggests that an increase in CLR, mediated by BMI, decreases TBS and reduces the risk of osteoporosis. However, it was found that too high a CLR had a significant detrimental effect on the microstructure of bone trabeculae, as assessed by TBS. Meanwhile, we also suggest incorporating TBS into routine bone health assessments. Future studies should investigate longitudinal changes in CLR and TBS to clarify causality in the relationship with BMI-mediated osteoporosis.

## Acknowledgments

We gratefully acknowledge the support from Wangjing Hospital, China Academy of Chinese Medical Sciences, and appreciate the reviewers’ valuable feedback, which has improved this manuscript. All authors express their gratitude to all participants and personnel involved in the NHANES.

## Author contributions

**Conceptualization:** Zhizhuang Wang, Bo Xu, Guoliang Ma.

**Data curation:** Zhizhuang Wang, Bo Xu.

**Formal analysis:** Bo Xu, Guoliang Ma.

**Funding acquisition:** Liguo Zhu, He Yin.

**Investigation:** Xin Chen, Bowen Yang, Xiaokuan Qin, Weili Duan, Minshan Feng, Kai Sun.

**Methodology:** Xin Chen, Bowen Yang, Xiaokuan Qin, Weili Duan, Minshan Feng, Kai Sun.

**Resources:** He Yin.

**Supervision:** Kai Sun, Liguo Zhu, He Yin.

**Writing – original draft:** Zhizhuang Wang.

## Supplementary Material



## References

[1] Lorentzon M Cummings SR. Osteoporosis: the evolution of a diagnosis. J Intern Med. 2015;277:650–61. 25832448 10.1111/joim.12369

[2] Rejnmark L Ejlsmark-Svensson H. Effects of PTH and PTH Hypersecretion on bone: a clinical perspective. Curr Osteoporos Rep. 2020;18:103–14. 32222892 10.1007/s11914-020-00574-7

[3] Wang CY Fu S-H Yang R-S Shen L-J Wu FL Hsiao F-Y. Age- and gender-specific epidemiology, treatment patterns, and economic burden of osteoporosis and associated fracture in Taiwan between 2009 and 2013. Arch Osteoporos. 2017;12:92. 29067572 10.1007/s11657-017-0385-5

[4] Kanters TA van de Ree CLP de Jongh MAC Gosens T Hakkaart-van Roijen L. Burden of illness of hip fractures in elderly Dutch patients. Arch Osteoporos. 2020;15:11. 31897865 10.1007/s11657-019-0678-y PMC6940317

[5] Chandran M Lau TC Gagnon-Arpin I. The health and economic burden of osteoporotic fractures in Singapore and the potential impact of increasing treatment rates through more pharmacological options. Arch Osteoporos. 2019;14:114. 31773442 10.1007/s11657-019-0664-4

[6] Orwoll ES Lapidus J Wang PY. The limited clinical utility of testosterone, estradiol, and sex hormone binding globulin measurements in the prediction of fracture risk and bone loss in older men. J Bone Miner Res. 2017;32:633–40. 27753150 10.1002/jbmr.3021 PMC5896330

[7] Bliuc D Alarkawi D Nguyen TV Eisman JA Center JR. Risk of subsequent fractures and mortality in elderly women and men with fragility fractures with and without osteoporotic bone density: the Dubbo Osteoporosis Epidemiology Study. J Bone Miner Res. 2015;30:637–46. 25359586 10.1002/jbmr.2393

[8] Kim JH Choi HJ Ku EJ. Trabecular bone score as an indicator for skeletal deterioration in diabetes. J Clin Endocrinol Metab. 2015;100:475–82. 25368976 10.1210/jc.2014-2047

[9] Leslie WD Majumdar SR Morin SN Hans D Lix LM. Change in Trabecular Bone Score (TBS) with antiresorptive therapy does not predict fracture in women: the manitoba BMD cohort. J Bone Miner Res. 2017;32:618–23. 27933656 10.1002/jbmr.3054

[10] Bedimo RJ Adams-Huet B Poindexter J. The differential effects of human immunodeficiency virus and hepatitis C virus on bone microarchitecture and fracture risk. Clin Infect Dis. 2018;66:1442–7. 29145609 10.1093/cid/cix1011

[11] Gonzalez Rodriguez E Lamy O Stoll D. High evening cortisol level is associated with low TBS and increased prevalent vertebral fractures: osteolaus study. J Clin Endocrinol Metab. 2017;102:2628–36. 28379565 10.1210/jc.2016-3804

[12] Karimi A Shobeiri P Kulasinghe A Rezaei N. Novel systemic inflammation markers to predict COVID-19 prognosis. Front Immunol. 2021;12:741061. 34745112 10.3389/fimmu.2021.741061 PMC8569430

[13] Fan Z Luo G Gong Y. Prognostic value of the C-reactive protein/lymphocyte ratio in pancreatic cancer. Ann Surg Oncol. 2020;27:4017–25. 32144621 10.1245/s10434-020-08301-3

[14] Kilinç EA Varkal G Kirmizier G Türk I Özer HTE. Are monocyte-to-HDL and C-reactive protein-to-albumin ratios useful for the diagnosis and follow-up of Takayasu arteritis? Rev Assoc Med Bras (1992). 2024;70:e20231683. 38775535 10.1590/1806-9282.20231683 PMC11101177

[15] Shao YJ Yu GD Zhang X Ran YG Li JH. Prognostic value of lymphocyte–C-reactive protein ratio in patients with liver cancer: a meta-analysis. Biomark Med. 2023;17:497–507. 37526144 10.2217/bmm-2023-0270

[16] Syauqy A Hsu C-Y Rau H-H Chao JC-J. Association of dietary patterns, anthropometric measurements, and metabolic parameters with C-reactive protein and neutrophil-to-lymphocyte ratio in middle-aged and older adults with metabolic syndrome in Taiwan: a cross-sectional study. Nutr J. 2018;17:106. 30454030 10.1186/s12937-018-0417-z PMC6240947

[17] Safari A Borhani-Haghighi A Dianatpour M Heydari ST Foroughinia F Ranjbar Omrani G. Circulating serum amyloid A, hs-CRP and vitamin D levels in postmenopausal osteoporosis. Galen Med J. 2019;8:e1548. 34466525 10.31661/gmj.v8i0.1548 PMC8343903

[18] Anyżewska A Łakomy R Lepionka T. Association between diet, physical activity and body mass index, fat mass index and bone mineral density of soldiers of the polish air cavalry units. Nutrients. 2020;12:242. 31963454 10.3390/nu12010242 PMC7019523

[19] Fujii N Tsukamoto M Okimoto N. Differences in the effects of BMI on bone microstructure between loaded and unloaded bones assessed by HR-pQCT in Japanese postmenopausal women. Osteoporosis Sarcopenia. 2021;7:54–62. 34278000 10.1016/j.afos.2021.05.002 PMC8261728

[20] Ilich JZ Gilman JC Cvijetic S Boschiero D. Chronic stress contributes to osteosarcopenic adiposity via inflammation and immune modulation: the case for more precise nutritional investigation. Nutrients. 2020;12:989. 32252359 10.3390/nu12040989 PMC7230299

[21] Chaitoff A Niforatos JD Gong J Fischer MA. A comparison of individuals with diabetes and EMPA-REG trial participants: exploring aspects of external validity. J Gen Intern Med. 2022;37:2744–50. 35031947 10.1007/s11606-021-07284-5 PMC9411404

[22] Ahn HJ Ishikawa K Kim MH. Exploring the diagnostic performance of machine learning in prediction of metabolic phenotypes focusing on thyroid function. PLoS One. 2024;19:e0304785. 38941283 10.1371/journal.pone.0304785 PMC11213305

[23] Ouyang Y Quan Y Guo C. Saturation effect of body mass index on bone mineral density in adolescents of different ages: a population-based study. Front Endocrinol (Lausanne). 2022;13:922903. 35865310 10.3389/fendo.2022.922903 PMC9294630

[24] Lobo PC de Branco FM Pichard C de Oliveira EP Pimentel GD. C-reactive protein, but not neutrophil–lymphocyte ratio, is inversely associated with muscle strength only in older men: NHANES 1999–2002. Exp Gerontol. 2023;173:112084. 36634720 10.1016/j.exger.2023.112084

[25] Li Y Wei Q Ke X. Higher CALLY index levels indicate lower sarcopenia risk among middle-aged and elderly community residents as well as hospitalized patients. Sci Rep. 2024;14:24591. 39426987 10.1038/s41598-024-75164-z PMC11490578

[26] Yan H Wang S Cao H Zhong H Sun C. The study findings demonstrated a significant association between C-reactive protein levels and trabecular bone score: NHANES 2005-2008. J Orthop Surg Res. 2024;19:519. 39210439 10.1186/s13018-024-05014-1 PMC11360293

[27] McCarty MF Lewis Lujan L Iloki Assanga S. Targeting Sirt1, AMPK, Nrf2, CK2, and soluble guanylate cyclase with nutraceuticals: a practical strategy for preserving bone mass. Int J Mol Sci . 2022;23:4776. 35563167 10.3390/ijms23094776 PMC9104509

[28] Kim JH Choi HJ Ku EJ. Regional body fat depots differently affect bone microarchitecture in postmenopausal Korean women. Osteoporos Int. 2016;27:1161–8. 26475286 10.1007/s00198-015-3329-1

[29] Wood IS Wang B Jenkins JR Trayhurn P. The pro-inflammatory cytokine IL-18 is expressed in human adipose tissue and strongly upregulated by TNFalpha in human adipocytes. Biochem Biophys Res Commun. 2005;337:422–9. 16188228 10.1016/j.bbrc.2005.09.068

[30] Pou KM Massaro JM Hoffmann U. Visceral and subcutaneous adipose tissue volumes are cross-sectionally related to markers of inflammation and oxidative stress: the Framingham Heart Study. Circulation. 2007;116:1234–41. 17709633 10.1161/CIRCULATIONAHA.107.710509

[31] Navarro MC Sosa M Saavedra P. Poverty is a risk factor for osteoporotic fractures. Osteoporos Int. 2009;20:393–8. 18773136 10.1007/s00198-008-0697-9

[32] Chen Z Ye H Li E Lin Y Jin C Yang L. Lipid accumulation product, poverty income ratio, and bone mineral density in U.S. adults: a mediation analysis based on NHANES (2009–2020). Front Nutr. 2024;11:1466288. 39421618 10.3389/fnut.2024.1466288 PMC11484405

[33] Zhang Y Tan C Tan W. BMI, socioeconomic status, and bone mineral density in U.S. adults: Mediation analysis in the NHANES. Front Nutr. 2023;10:1132234. 36960203 10.3389/fnut.2023.1132234 PMC10027781

[34] Crandall CJ Merkin SS Seeman TE Greendale GA Binkley N Karlamangla AS. Socioeconomic status over the life-course and adult bone mineral density: the Midlife in the U.S. Study. Bone. 2012;51:107–13. 22543227 10.1016/j.bone.2012.04.009 PMC3371160

[35] Torgutalp SS Babayeva N Kara OS Özkan O Dönmez G Korkusuz F. Trabecular bone score of postmenopausal women is positively correlated with bone mineral density and negatively correlated with age and body mass index. Menopause. 2019;26:1166–70. 31188287 10.1097/GME.0000000000001375

[36] Shin YH Gong HS Lee KJ Baek GH. Older age and higher body mass index are associated with a more degraded trabecular bone score compared to bone mineral density. J Clin Densitom. 2019;22:266–71. 28712983 10.1016/j.jocd.2017.06.006

[37] Ellulu MS Khaza’ai H Rahmat A Patimah I Abed Y. Obesity can predict and promote systemic inflammation in healthy adults. Int J Cardiol. 2016;215:318–24. 27128554 10.1016/j.ijcard.2016.04.089

[38] Al Khathlan N. Association of inflammatory cytokines with obesity and pulmonary function testing. PLoS One. 2023;18:e0294592. 37992066 10.1371/journal.pone.0294592 PMC10664933

[39] Mohamed GA Abd-Elrahman MZ Bahriz R Albehairy A. Inflammatory cytokine and plasma C-reactive protein response to ketoacidosis in adults with type 1 diabetes: Egyptian multicenter study. Egypt J Internal Med. 2020;32:10.

[40] Landecho MF Tuero C Valentí V Bilbao I de la Higuera M Frühbeck G. Relevance of leptin and other adipokines in obesity-associated cardiovascular risk. Nutrients. 2019;11:2664. 31694146 10.3390/nu11112664 PMC6893824

[41] Poosri S Vimaleswaran KS Prangthip P. Dietary lipids shape cytokine and leptin profiles in obesity-metabolic syndrome implications: a cross-sectional study. PLoS One. 2024;19:e0315711. 39700087 10.1371/journal.pone.0315711 PMC11658627

[42] Ağbaht K Gürlek A Karakaya J Bayraktar M. Circulating adiponectin represents a biomarker of the association between adiposity and bone mineral density. Endocrine. 2009;35:371–9. 19288226 10.1007/s12020-009-9158-2

[43] Olson ME Hornick MG Stefanski A. A biofunctional review of C-reactive protein (CRP) as a mediator of inflammatory and immune responses: differentiating pentameric and modified CRP isoform effects. Front Immunol. 2023;14:1264383. 37781355 10.3389/fimmu.2023.1264383 PMC10540681

[44] Riou R Bressollette-Bodin C Boutoille D. Severe symptomatic primary human cytomegalovirus infection despite effective innate and adaptive immune responses. J Virol. 2017;91:e02245-16. 28031361 10.1128/JVI.02245-16 PMC5309965

[45] Cheng S Lyu J Shi X Wang K Wang Z. Rare variant association tests for ancestry-matched case–control data based on conditional logistic regression. Brief Bioinform. 2022;23:1–12. 10.1093/bib/bbab572 35021184

[46] Li D Liu J Guo B. Osteoclast-derived exosomal miR-214-3p inhibits osteoblastic bone formation. Nat Commun. 2016;7:10872. 26947250 10.1038/ncomms10872 PMC4786676

[47] Zhao Z Du Y Yan K Zhang L Guo Q. Exercise and osteoimmunology in bone remodeling. FASEB J. 2024;38:e23554. 38588175 10.1096/fj.202301508RRR

[48] Shen L Zong Y Zhao J. Characterizing the skeletal muscle immune microenvironment for sarcopenia: insights from transcriptome analysis and histological validation. Front Immunol. 2024;15:1414387. 39026669 10.3389/fimmu.2024.1414387 PMC11254692

[49] Molina P Carrero JJ Bover J Chauveau P Mazzaferro S Torres PU. Vitamin D, a modulator of musculoskeletal health in chronic kidney disease. J Cachexia Sarcopenia Muscle. 2017;8:686–701. 28675610 10.1002/jcsm.12218 PMC5659055

[50] Slowicka K Vereecke L van Loo G. Cellular functions of optineurin in health and disease. Trends Immunol. 2016;37:621–33. 27480243 10.1016/j.it.2016.07.002

[51] Tsukasaki M Takayanagi H. Osteoimmunology: evolving concepts in bone–immune interactions in health and disease. Nat Rev Immunol. 2019;19:626–42. 31186549 10.1038/s41577-019-0178-8

