# Supplementary figures and images for: C-reactive protein-to-lymphocyte ratio and trabecular bone score: A mediation analysis of BMI in the NHANES 2005 to 2008 cohort
Source: Medicine (Baltimore). 2025 Aug 8;104(32):e43847. doi: 10.1097/MD.0000000000043847 (PMC12338286; doi:10.1097/MD.0000000000043847)

Supplementary Material

## 1.Ethical date and lot number


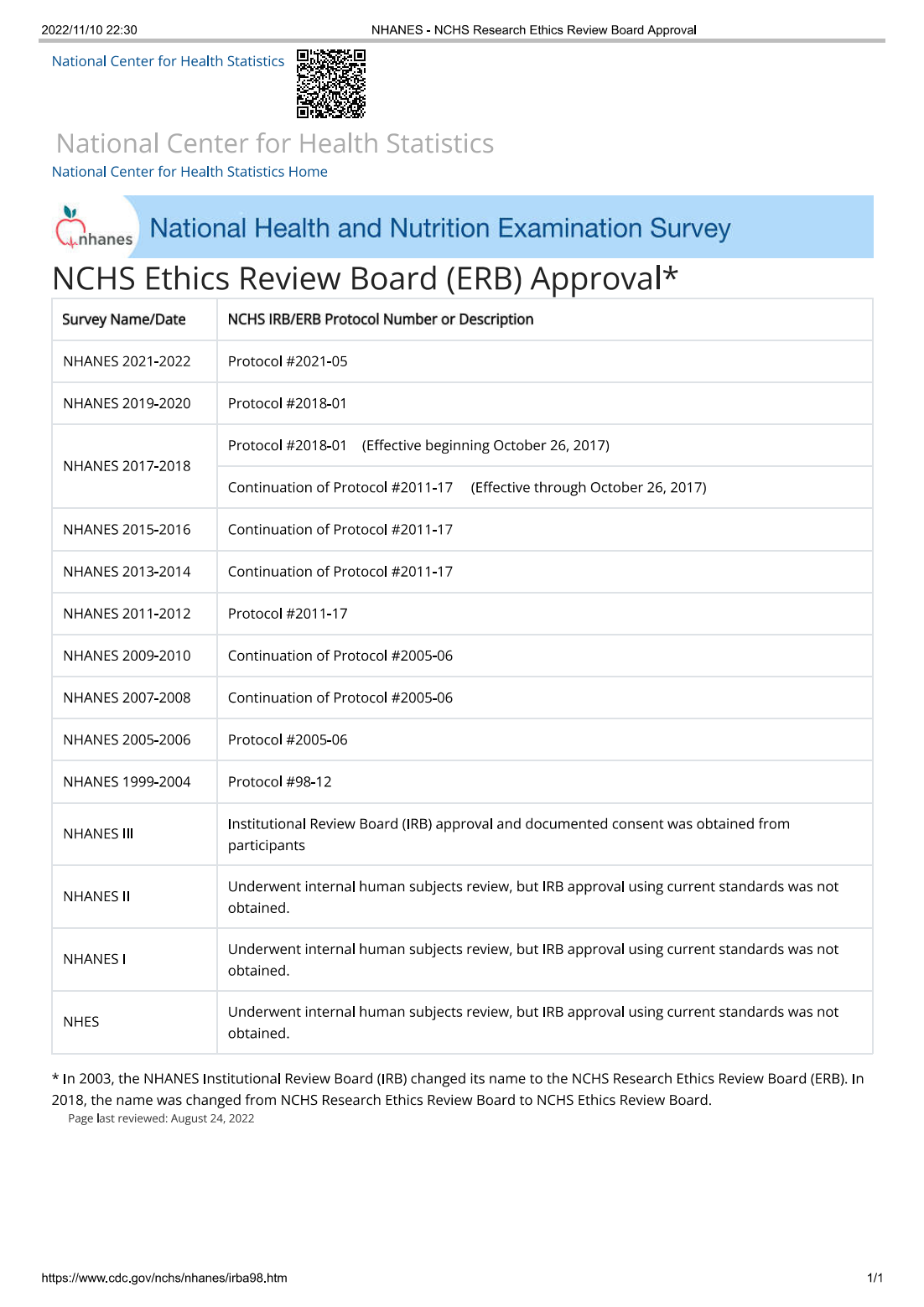

Supplement: Supplementary file 1 [file medi-104-e43847-s001.docx]
